# Protonation and deprotonation induced organo/hydrogelation: Bile acid derived gelators containing a basic side chain

**DOI:** 10.3762/bjoc.7.40

**Published:** 2011-03-10

**Authors:** Uday Maitra, Arkajyoti Chakrabarty

**Affiliations:** 1 Department of Organic Chemistry, Indian Institute of Science, Bangalore-560012, India

**Keywords:** bile acid derived amines, organogelator and hydrogelator, protonation and deprotonation induced gelation, SEM and POM, thermal stability

## Abstract

Two bile acid derived molecules containing basic amino groups are reported to be efficient and unusual gelators of organic and aqueous solvents.

## Introduction

Low molecular mass organo- and hydrogelators (LMOG) have attracted considerable attention in recent years due to their tunable physical properties, such as stimuli sensitivity. Their self-assembly in nanoscale superstructures are likely to have important implications in accessing functional nanomaterials [1]. The types of superstructures generated by the SAFINs (Self-Assembled Fibrillar Networks) include fibres, rods, and ribbons. Such self-assembled structures form mainly due to weak non-covalent interactions such as hydrogen-bonding, van der Waals forces, π–π interactions, charge-transfer interactions etc. in organogels, whereas, in aqueous gels, the major driving force for aggregation is hydrophobic interaction [2]. A number of hydrogelators derived from the bile acid backbone have been described in the literature [3–5]. The earliest reports include sodium deoxycholate which forms a gel in water at pH 6.9 [6] and calcium cholate which gels water at pH 7 [7–9]. The facial amphiphilicity of the bile acid derivatives appears to be primarily responsible for their aggregation in water. Unlike conventional surfactant molecules, bile acid salts possess a rigid steroidal backbone, with polar hydroxyl groups on the concave α-face and methyl groups on the convex β-face. On the other hand, relatively few bile acid derived organogelators have been reported [10–15]. Our group has previously reported charge-transfer interaction driven organogelation based on bile acid derived, and other donor molecules [16].

**Scheme 1:**
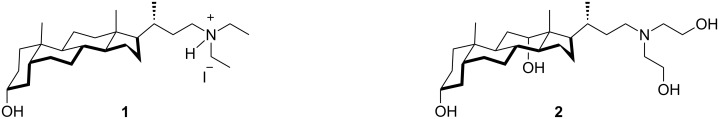
Gelators **1** and **2**.

The present work describes efficient organo/hydrogelation by two bile acid-derived low molecular mass gelators **1** and **2** (Scheme 1) having remarkably simple structures with amino groups in the side-chain.

## Results and Discussion

**Figure 1:**
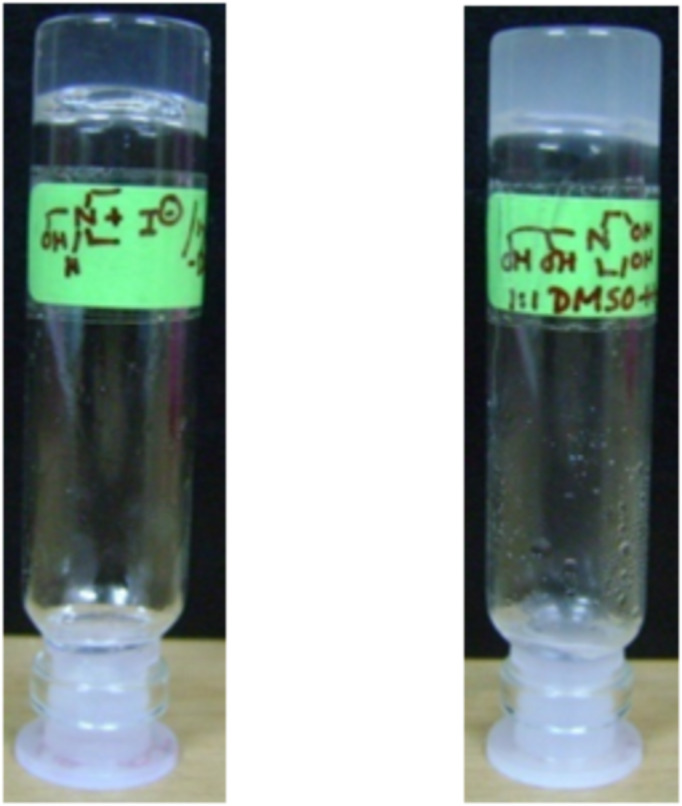
Photographs of the gels: **1** in 1,2-dichlorobenzene (0.2% w/v, left); **2** in 1:1 DMSO/water (0.3% w/v, right).

Compound **1** was found to be a super gelator of organic solvents such as 1,2-dichlorobenzene and chlorobenzene and gelled these solvents at very low concentrations (0.05% w/v). In contrast, **2** was found to gel mixtures of aqueous organic solvents such as DMSO/water and DMF/water. Interestingly, it is the protonated amine **1** which has the organogelation property; whilst **2** must be in the neutral form for hydrogelation (Figure 1).

### (A) Gelation behaviour of **1** and **2**

The gelation tests were carried out with compounds **1** and **2** in various organic and mixtures of aqueous organic solvents (Experimental section); the results of the gelation studies are summarized in Table 1.

**Table 1:** Gelation behaviour of 1 and 2^a^.

	1	2

Chloroform	S	S
Mesitylene	S	P
1,2-Dichlorobenzene	TG (CGC 2 mM)	TG (W)
Chlorobenzene	TG (CGC 2 mM)	GP
Benzene	I	P
Toluene	I	GP
Isopropanol	S	S
DMSO/water	P	TG (CGC 5 mM)^b^
DMF/water	S	TG
MeOH/water	P	GP
AcOH/water	S	S
Acetone/water	S	GP
Dioxane/water	S	TLG
CH_3_CN/water	S	OG
Water	I	I

^a^TG, transparent gel; TLG, translucent gel; GP, gelatinous precipitate; S, solution; I, insoluble; P, precipitate; OG, opaque gel; W, weak. ^b^**2** formed gel in mixtures of DMSO/water (1:2 to 3:2), DMF/water (2:3 to 3:2), 1,4-dioxane/water (1:4) and acetonitrile/water (1:3).

### (B) Protonation–deprotonation induced gelation

The organogelator **1** was found to be a non-gelator in its neutral form, whereas when it was used as its iodide salt it formed strong gels in 1,2-dichlorobenzene and chlorobenzene. To illustrate the acid-base switching of this gel, a simple experiment was designed to show the reversible switching from gel→sol→gel of **1** in 1,2-dichlorobenzene using cresol red sodium salt as the acid–base indicator.

**Figure 2:**
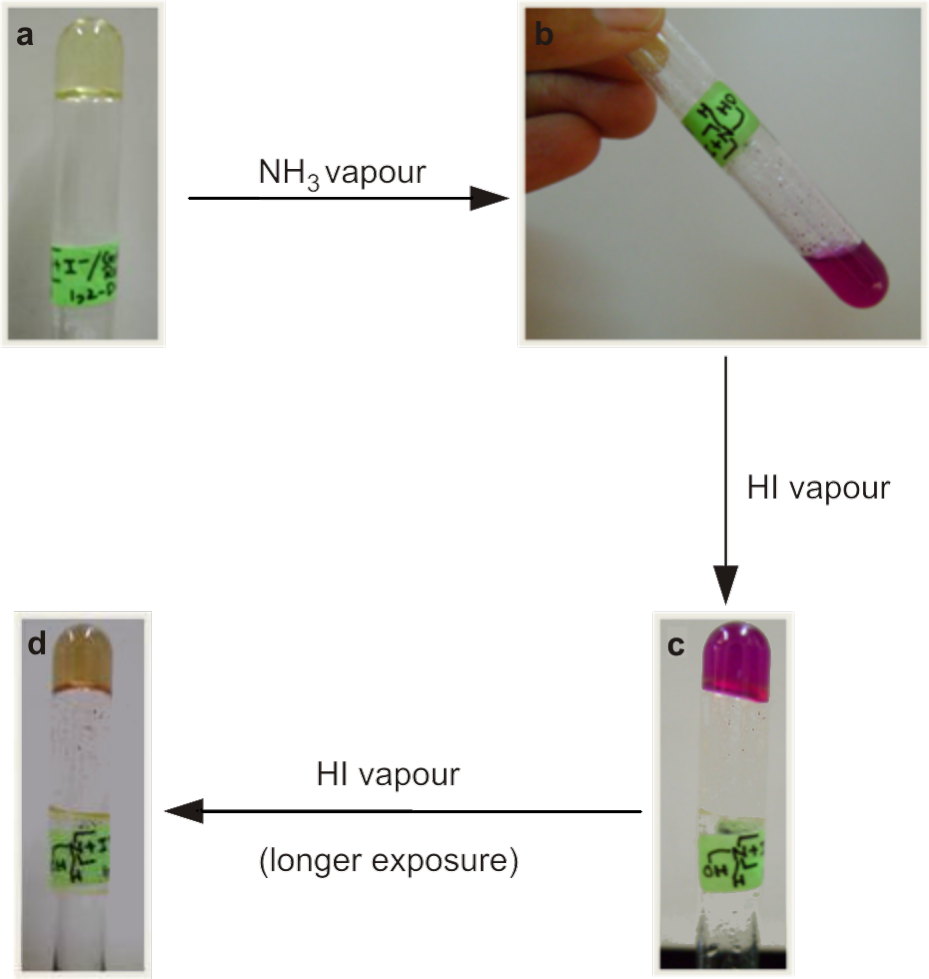
Illustration of base-instability and acid-stability of the organogel of **1** in 1,2-dichlorobenzene.

Upon exposure to ammonia vapour, the gel (Figure 2a) formed a solution (Figure 2b, the solution did not form a gel on heating and cooling) with a concomitant colour change (yellow to pink). When this pink solution was exposed to HI vapour, the gel was reformed (Figure 2c, heating was required to dissolve the salt formed and the gel formed upon cooling to room temperature) with the colour turning yellow again (Figure 2d). It is important to note that cresol red turns yellow in water below pH 7.2 and pink above pH 8.8 [17–18]. If the pink solution (Figure 2b) was heated at 120 °C and exposed to HI vapour (this was done by keeping the test tube containing the hot solution inside a sealed chamber containing conc. HI), sol to gel conversion did not require further heating and cooling.

**Figure 3:**
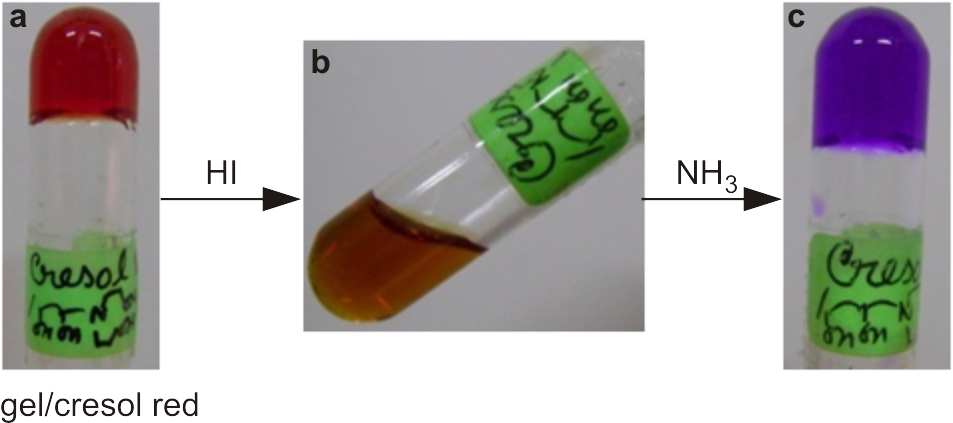
Acid-instability and base-stability of the hydrogel of **2** in 1:1 DMSO/water.

However, for the hydrogel derived from **2**, the situation was reversed. The neutral amine **2** formed a gel in 1:1 DMSO/water (0.5% w/v). When the gel was doped with cresol red, it developed a red colour (Figure 3a), indicating a “pH” of 7.2. However, when 10 μl of HI (conc. HI (7 M, 57%) was diluted 20-fold and ~0.6 equiv of acid was used with respect to the amine) was added to the gel, the gel framework was disrupted and the solution turned yellow (Figure 3b, the gel did not reform upon heating and cooling/sonication) indicating the solution has “pH” <7.2. The addition of 10 μl of 25% aq. ammonia (13 M, ~30 equiv of ammonia was used with respect to the protonated amine) triggered the sol to gel transition and this time the gel turned pink colour (Figure 3c, heating and cooling reformed the gel).

### (C) SEM and POM characterization of the gels

**Figure 4:**
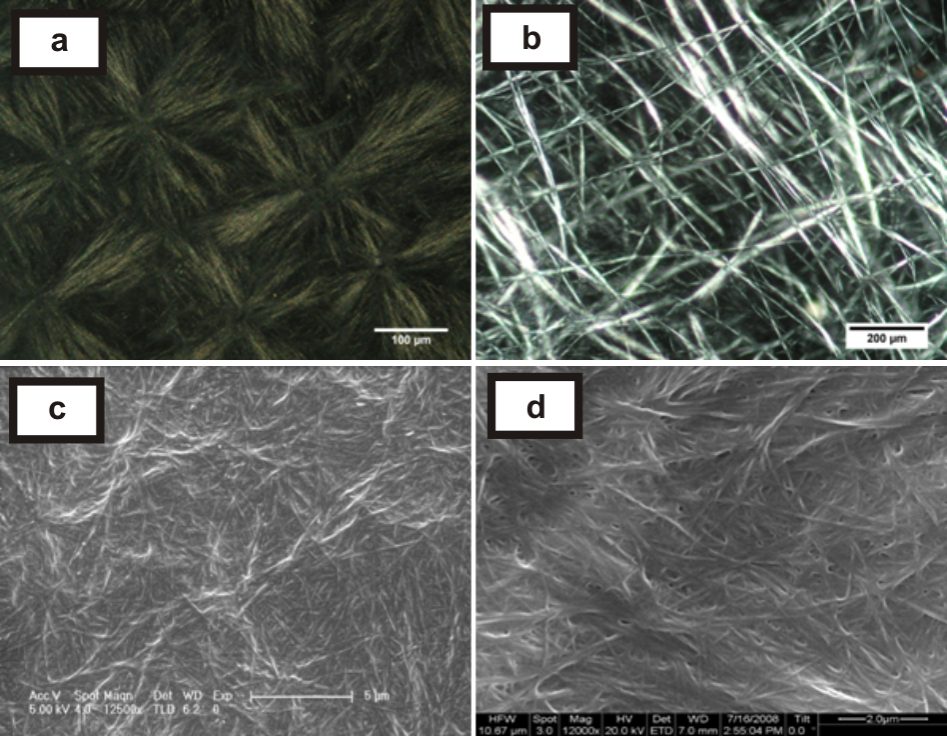
(a) and (b) POM images of the gels of diethylaminolithocholyl iodide **1** in 1,2-dichlorobenzene (1.25 and 0.25% w/v of gelator, respectively); (c) and (d) SEM images of xerogels of **1** in 1,2-dichlorobenzene (0.5 and 1% w/v, respectively).

The gels showed birefringent textures under a polarizing optical microscope [19]. The organogel showed spherulitic structures [20] (where the fibres originated from nucleation centres, Figure 4a) and a highly entangled fibrillar network (Figure 4b) at higher (1.25% w/v) and lower (0.25% w/v) concentrations of gelator, respectively. SEM images showed the presence of fine fibres (diameter <1μm) in the organogel (Figure 4c, Figure 4d).

**Figure 5:**
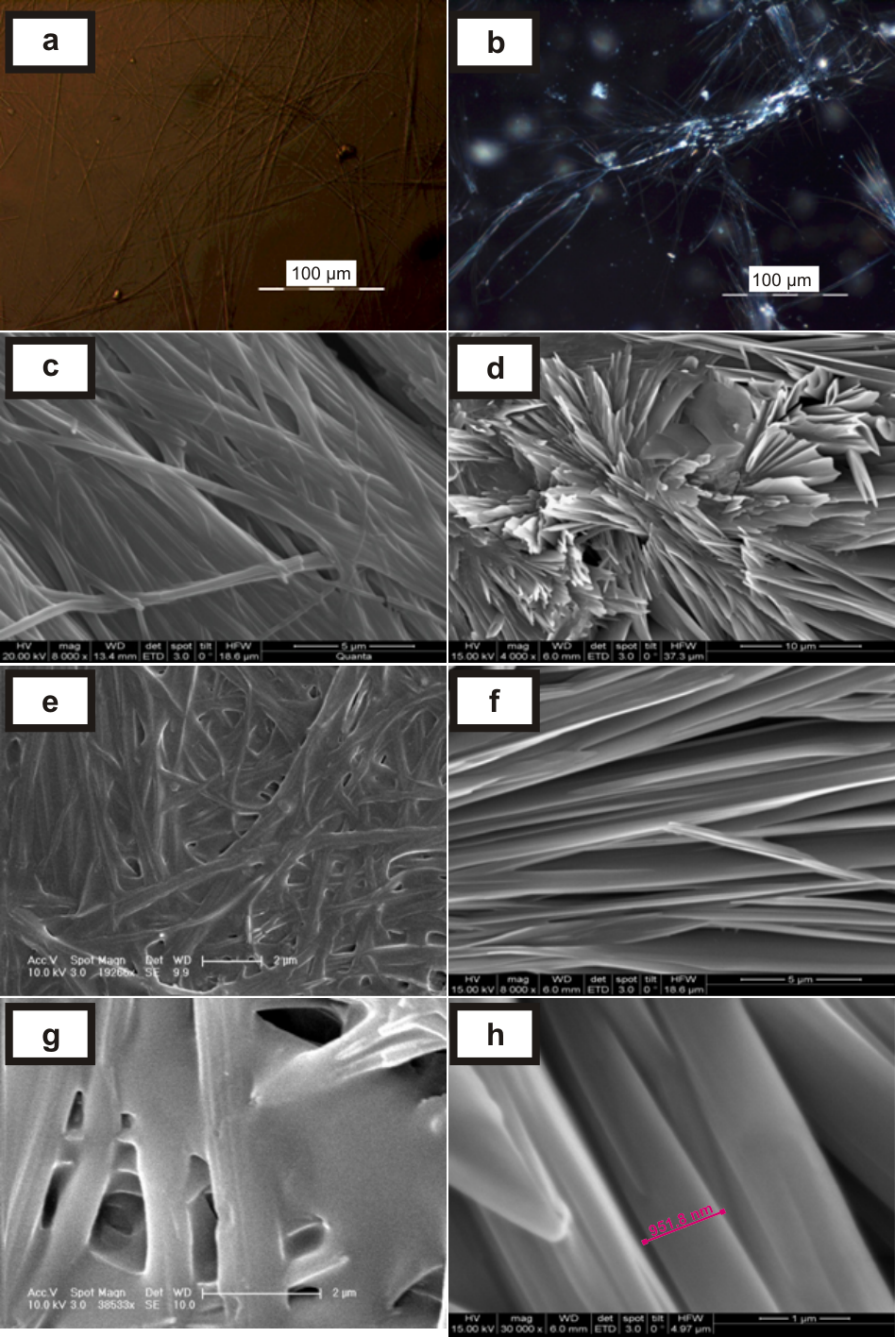
(a) and (b) POM images of bis(2-hydroxyethyl)aminodeoxycholane **2** gel in 1:1 DMSO/water (normally-cooled gel); (c), (e) and (g) SEM images of the xerogels (normally cooled gels); (d), (f) and (h) SEM images of the xerogels (heated and sonicated).

However, for the DMSO/water hydrogel (normally cooled), inter-connected fibres (Figure 5a) and some needle-like microcrystallites (Figure 5b) were observed under a polarizing optical microscope. Interestingly, there were two types of morphology observed in the SEM micrographs: Normally-cooled gels showed finer fibres as compared to the sonication-induced gel. The arrangement of the fibres were found to be different in the normally cooled gel (Figure 5c, 5e, 5g) in comparison to the sonication induced gel (Figure 5d, 5f, 5h) [21].

### (D) Thermal stability of the gels

**Figure 6:**
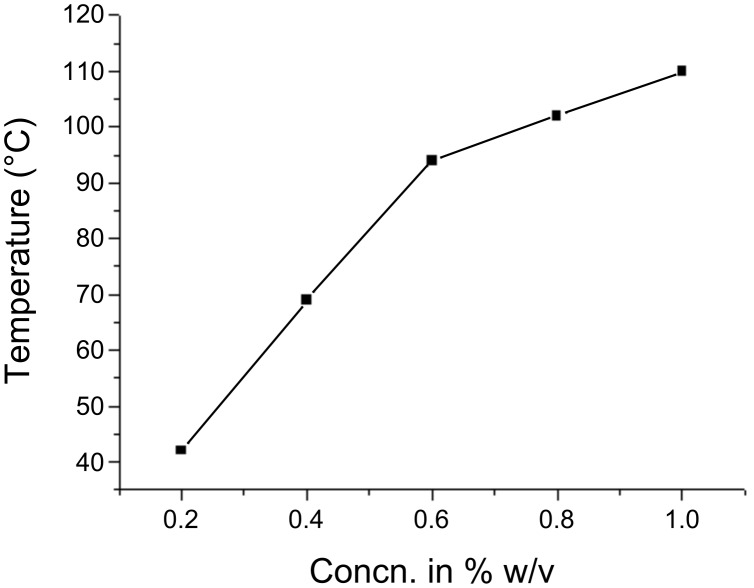
Gel melting profile of diethylaminolithocholyl iodide **1** gel in 1,2-dichlorobenzene.

The concentration dependence of the thermal stability of **1**/1,2-dichlorobenzene gel was carried out by the “inverted test-tube” method [22]. The sharp increase in melting point of the gels (Figure 6) containing 0.2 to 0.6% w/v of gelator could be due to the maximal interaction between solvent and gelator molecules leading to gelation [23]. There were also observable changes in the POM images as the gelator concentration was varied from 0.25 to 1.25% w/v (Figure 4a and 4b).

**Figure 7:**
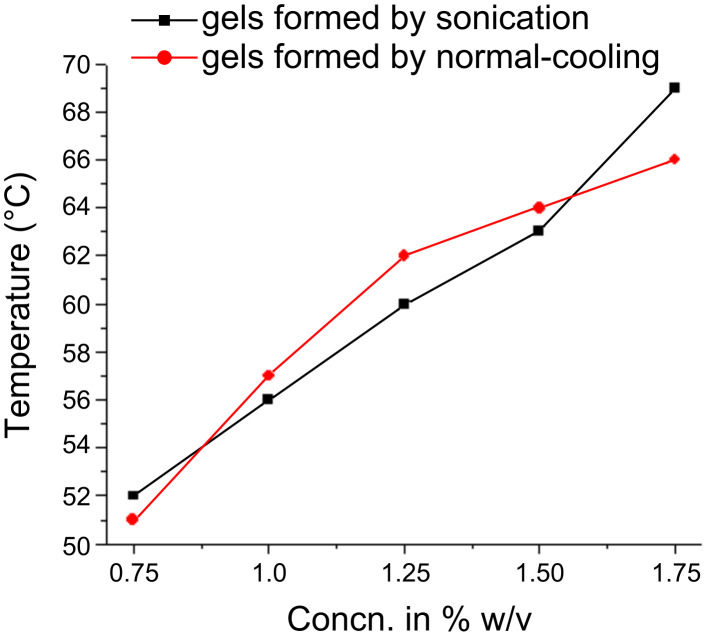
Gel melting profile of bis(2-hydroxyethyl)aminodeoxycholane **2** gel in 1:1 DMSO/water (normally cooled (red) and sonication-induced (black) gels).

Thermal stability studies on the gels obtained from **2** in 1:1 DMSO/water (Figure 7) showed that normally cooled gels melted from 51–66 °C (gelator concentration 0.75 to 1.75% w/v, 12–38 mM). The melting profile of the sonicated samples was found to be very similar to that of the normally cooled gels. This suggests that while the sonication process after heating led to different structures of the SAFIN as illustrated in the SEM images, *thermal stabilities were unaffected.*

## Conclusion

In conclusion, we have demonstrated an interesting protonation and deprotonation induced gelation of an organogelator and a hydrogelator, respectively. Using cresol red as an indicator, it was possible to illustrate the acid-stability and base-instability of the organogel and the acid-instability and base-stability of the hydrogel.

However, it was also found that the organogel showed high thermal stability and the nanoscale morphology represented fibres of diameters ranging from 80 nm to 1 μm. The hydrogel had comparatively lower thermal stability and showed different morphologies on sonication induced gelation and normally cooled gelation phenomenon. The hydrogel consisted of fine fibres and birefringent textures when investigated under a polarizing optical microscope.

Finally, these low molecular mass gelators which gel organic and aqueous organic solvents, represent a new class of gelators which have the ability to respond to acid-base stimuli and are potentially useful in emerging fields [24–26].

## Experimental

### Materials

The syntheses of gelators were carried out starting from lithocholic and 7-deoxycholic acids supplied by Sigma. Diethylamine was purchased from Aldrich and diethanolamine was obtained from a local supplier. Solvents were distilled prior to use.

#### Instruments

Olympus BX 51 polarizing optical microscope was used for recording POM images of the gels. SEM images were recorded using E-SEM Quanta machine operating at 10–20 kV and xerogels were gold-coated with 10 nm thickness before recording images. For recording gel melting temperatures a Heidolph stirrer-heater was used and test tubes were sealed at the top after preparing the gels. The test tubes containing the gels were kept upside down in a water bath/paraffin oil bath. Temperature was increased at a controlled rate (~2 °C/min). The temperature at which the gels fell under gravity was noted as the gel melting temperatures. In preparing POM samples, the gels were carefully scooped up and placed over a clean microscope slide covering the sample with a thin cover slip. In case of SEM, scooped up gels were placed over carbon tapes pasted on aluminium stubs and allowed to dry at room temperature in a desiccator connected to vacuum pump.

### Brief synthetic procedure

**Scheme 2:**
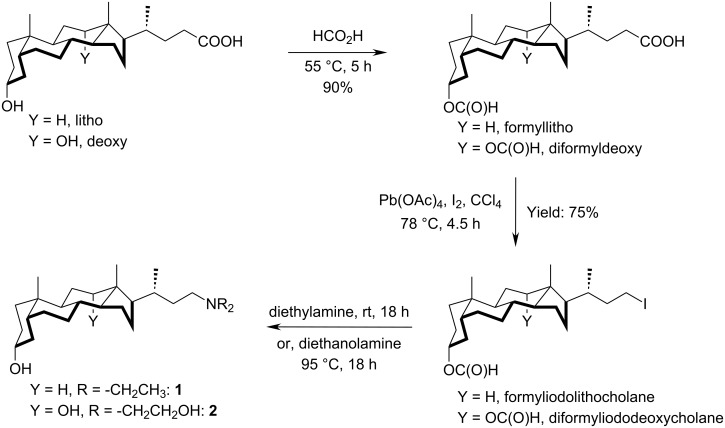
General method of synthesis of bile acid derived amines **1** and **2**.

Organogelator **1** and hydrogelator **2** were synthesized starting from lithocholic acid and deoxycholic acid, respectively, as shown in Scheme 2. Formylated lithocholic, deoxycholic acid and formyliodolithocholane, diformyliododeoxycholane were synthesized according to reported procedures [27–28].

#### Synthesis of compound **1**

3α-Formyloxy-5β-23-iodo-24-norcholane (0.50 g, 1.03 mmol) was dissolved in diethylamine (10 mL, 96 mmol) and stirred at 50 °C for 18 h. After removing the volatiles, the crude product was purified by column chromatography on silica gel (2.5 cm × 10.0 cm) with 5–10% EtOH/CHCl_3_ as eluent to yield 0.53 g (97%) of the salt. The product was re-precipitated with CHCl_3_/hexane (1:20) and separated by centrifugation. This process was repeated twice to obtain the pure salt (0.40 g, 74%). m.p. 272–276 °C. [α]_D_^24^: 36 (*c* 2.00, EtOH).^1^H NMR (400 MHz, CDCl_3_): δ 3.64 (m, 1H), 3.23–2.97 (br m, 6H), 2.03–1.52 (steroidal CH_2_), 1.48 (t, 6H, *J* = 7.2 Hz), 1.42–1.10 (steroidal CH_2_), 0.99 (d, 3H, *J* = 5.6 Hz), 0.92 (s, 3H), 0.65 (s, 3H). ^13^C NMR (75 MHz, CDCl_3_): δ 71.7, 56.4, 55.6, 49.4, 47.3, 42.8, 42.0, 40.3, 40.1, 36.4, 35.8, 35.3, 34.5, 34.3, 30.5, 28.8, 28.5, 27.1, 26.3, 24.1, 23.3, 20.7, 18.7, 12.0, 8.8. IR (KBr): 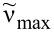 3457, 2927, 2860, 1457, 1040 cm^−1^. HRMS (ESI): Calcd. for C_27_H_50_NO^+^ [M + H]^+^ 404.3887; Found 404.3892. Anal. calcd. for C_27_H_50_NOI: C, 61.00, H 9.48, N, 2.63. Found: C, 61.14, H, 9.50, N, 3.10.

^1^H NMR of the neutral form of compound **1** i.e. C_27_H_49_NO showed the following pattern: ^1^H NMR (400 MHz, CDCl_3_): δ 3.63 (m, 1H), 2.57–2.36 (br m, 6H), 1.98–1.10 (steroidal CH_2_), 1.04 (t, 6H, *J* = 7.2 Hz), 0.94–0.92 (s, d merged, 6H), 0.64 (s, 3H).

#### Synthesis of compound **2**

3α,12α-Diformyloxy-5β-23-iodo-24-norcholane (0.74 g, 1.56 mmol) was stirred with diethanolamine (15 mL, 157 mmol) at 80 °C for 24 h. The reaction mixture was diluted with 150 mL of CHCl_3_ and washed with water (2 × 50 mL) in a separatory funnel (250 mL). The organic layer was dried over anhydrous Na_2_SO_4_. The crude product was purified by column chromatography on silica gel (2.5 cm × 10.0 cm) with 20–40% EtOH/CHCl_3_ as eluent. The column purified product was passed through a column of basic alumina (0.7 cm × 16 cm) to remove traces of acidic impurities. The neutral amine was obtained in 66% yield (0.46 g). m.p.: 155–156 °C. [α]_D_^29^: 44 (*c* 2.00, EtOH). ^1^H NMR (300 MHz, CDCl_3_): δ 3.98 (br s, 1H), 3.65–3.58 (m, 5H), 2.74–2.47 (m, 6H), 1.00 (d, *J* = 6.6 Hz, 3H), 0.91 (s, 3H), 0.68 (s, 3H). ^13^C NMR (75 MHz, CDCl_3_): δ 73.1, 71.7, 59.4, 56.1, 52.0, 48.2, 47.2, 46.5, 42.1, 36.4, 36.0, 35.2, 34.1, 33.6, 32.4, 30.4, 29.6, 28.6, 27.7, 27.1, 26.1, 23.6, 23.1, 17.9, 12.7. IR (KBr): 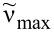 3375, 2935, 2863, 1470, 1448, 1045 cm^−1^. LRMS (ESI): Calcd. for C_27_H_49_NO_4_Na 474. Found 474. Anal. calcd. for C_27_H_49_NO_4_: C, 71.80, H, 10.93, N 3.10. Found: C, 71.45, H, 10.82, N, 3.20.

### Gelation procedure

The gelation tests were performed by dissolving compound **1** in 1,2-dichlorobenzene by heating at 120 °C and then allowing to cool to room temperature to form the gel. The gels formed very fast (2–15 min depending upon the gelator concentration). A translucent gel formed when the gelator **2** was dissolved in DMSO followed by addition of water at rt. If the resulting solution was heated at 110 °C to yield a transparent solution, it took 5–10 min to form an almost transparent gel upon cooling to rt. However, a transparent gel was obtained when the hot solution was sonicated for 35–40 s.
